# Physical Activity and Nutritional Supportive Care Needs Following a Sarcoma Diagnosis: Barriers and Opportunities for Rare Cancer Survivorship Care

**DOI:** 10.1002/pon.70567

**Published:** 2026-08-17

**Authors:** Georgia K. B. Halkett, Jenny Davies, Chloe Maxwell‐Smith, Mandy Basson, Tania Rice‐Brading, Mariana S. Sousa, Janene Sproul, Helen De Jong, Haryana M. Dhillon, Connor Farnell, William Lorimer, Joanna Fardell, Joanne Shaw, Moira O'Connor

**Affiliations:** ^1^ Curtin School of Nursing Faculty of Health Sciences Curtin University Perth Western Australia Australia; ^2^ Faculty of Health Sciences Curtin Medical Research Institute Curtin University Perth Western Australia Australia; ^3^ Curtin School of Population Health Curtin University Perth Western Australia Australia; ^4^ The Abbie Basson Sarcoma Foundation Ltd. Sock it to Sarcoma! Perth Western Australia Australia; ^5^ Cooper Rice‐Brading Foundation Sydney New South Wales Australia; ^6^ Faculty of Health Improving Palliative, Aged and Chronic Care Through Clinical Research and Translation (IMPACCT) University of Technology Sydney New South Wales Australia; ^7^ School of Education Murdoch University Perth Western Australia Australia; ^8^ Curtin School of Allied Health Curtin University Perth Western Australia Australia; ^9^ Occupational Therapy South Metropolitan Health Service Fiona Stanley Fremantle Hospital Group Perth Western Australia Australia; ^10^ School of Psychology Faculty of Science Psycho‐oncology Cooperative Research Group (PoCoG) The University of Sydney Sydney New South Wales Australia; ^11^ School of Clinical Medicine Faculty of Medicine and Health UNSW Sydney New South Wales Australia

**Keywords:** lifestyle factors, nutrition, physical activity, psycho‐oncology, qualitative research, recovery, sarcoma, supportive cancer care

## Abstract

**Background:**

People with primary bone and soft tissue sarcoma are a unique and vulnerable population at high risk of physical dysfunction and disability. Treatment often leads to complex challenges, including pain, limited mobility, and loss of function. Exercise has the potential to improve strength and function, reduce disability, and improve quality of life. Nutrition plays a crucial role in supporting recovery and immune function, and enhancing treatment outcomes. Yet, limited research has explored physical activity and nutritional needs of people with sarcoma.

**Aim:**

To explore the physical activity and nutritional supportive care needs of people living with sarcoma from the perspectives of people with sarcoma, their carers, and HCPs.

**Method:**

A phenomenological approach was employed to understand lived experiences of people with sarcoma and their carers focusing on what physical activity and nutritional supportive care people received. Semi‐structured interviews and focus groups were conducted with people with sarcoma, carers, and healthcare professionals. Multiple perspectives were included to understand the current clinical landscape for people experiencing sarcoma and healthcare professionals. Reflexive thematic analysis was used to draw meaning from the data and information power informed sample size.

**Results:**

We interviewed 46 participants (people with sarcoma *n* = 18, current carers *n* = 10, bereaved carers *n* = 3, and healthcare professionals *n* = 15) who emphasised the need for more information and support for physical activity and nutrition. Five themes were identified: *Physical impact of sarcoma*; *Uncertainty about looking after the person with sarcoma*; *Needing rehabilitation* *support; Creating a new norma*l; and, *Physical activity and healthy diet promote psychosocial well‐being*.

**Conclusion:**

As sarcoma substantially impacts physical functioning, it is essential that sarcoma‐specific physical activity and nutrition information and supports are available and tailored to individuals. Multidisciplinary teams should provide ongoing physical activity and nutritional supportive care from diagnosis and throughout survivorship to improve health outcomes and QoL for people with sarcoma.

## 1 Introduction

Sarcoma is a rare cancer arising from the bone, cartilage, or soft tissues [1]. Soft tissue sarcoma develops in the connective tissues that support organs, while bone sarcoma arises from bone and cartilage around joints. In Australia, approximately 2695 new cases of sarcoma are diagnosed each year [2]. Sarcomas account for 9.2% of childhood cancers, 8.8% of cancers in adolescents and young adults, and 1.5% of cancer diagnoses in adults [2]. Treatment for sarcoma can include surgery, radiation therapy, chemotherapy, and immunotherapy, depending on, tumour type, location, staging, and prognosis [3].

Sarcoma can lead to a wide range of physical symptoms that, coupled with aggressive treatments, place individuals at high risk of physical dysfunction and significant limitations [4]. These include pain, fatigue, bone weakness, restricted range of motion, loss of muscle strength, and reduced mobility, along with changed appearance impacting body image [4]. Following treatment, individuals may need emotional support to regain confidence and adjust to changes in their bodies and physical abilities [5, 6].

When sarcoma occurs in the limbs, the effects may be more pronounced. People with sarcoma may require limb‐sparing surgery or amputation, leading to restricted mobility and difficulty engaging in physical activity either from amputation or loss of muscle strength [5, 7]. Additionally, side effects of treatment can have long‐term impacts on patients' overall functionality and quality of life (QoL). After treatment for lower‐limb sarcoma, individuals may experience muscle atrophy and deconditioning due to their sedentary state [8, 9, 10]. Limb‐sparing surgery increases rehabilitation challenges, including multiple surgeries (reconstructions and/or revisions) and wound healing precautions to prevent infection limiting physical activity and access to equipment [11, 12]. For some, physical deficits severely compromise their autonomy, leading to dependence on informal carers.

Physical activity is an important component of cancer recovery, as it reduces treatment side effects and improves health outcomes [7, 13]. Physical activity improves muscle strength, bone strength, balance, and energy levels, and reduces progression of solid tumours [5, 14]. Physical activity also improves post‐surgery functionality and QoL [3, 7, 15].

Nutrition plays a vital role in cancer recovery and is a useful adjunct to physical activity in post‐treatment cancer care [16]. Cancer and its treatment can lead to loss of taste and appetite, nausea, vomiting, and gastrointestinal issues such as diarrhoea and/or constipation. Consequently, many people experience weight loss [14, 17] and 40%–80% of all people with cancer are malnourished during cancer treatment [18]. Malnutrition is a negative predictor for recovery [19] as it can affect treatment outcomes, delay wound healing, impair muscle function, and elevate the risk of post‐operative complications [18, 20].

Malnutrition and sarcopenia (muscle wasting and reduced strength and endurance) are associated with increased treatment toxicity, poorer survival outcomes and prolonged recovery [14, 16]. Malnutrition and sarcopenia are key clinical indicators prompting referral to nutrition and exercise physiology services in oncology, with the interplay between nutritional deficiency, muscle wasting, and impaired physical function being closely interconnected and highly relevant for this population [16]. Screening for malnutrition and sarcopenia is recommended at diagnosis and throughout treatment and recovery [16]. However, people with sarcoma may not be referred for support as required.

Engaging in healthy behaviours (including physical activity and adequate nutrition) during and following treatment for sarcoma can be challenging, due to physical, dietary, psychological, and social factors. Optimising physical recovery and QoL requires a focus on both physical activity and nutritional needs to minimise fatigue, muscle loss, cognitive impairment, and falls, increase confidence, and mitigate or reduce long‐term psychosocial issues.

Although the benefits of physical activity and adequate nutrition have been demonstrated for cancer generally, limited research has explored how people with sarcoma perceive their physical activity and nutritional needs. Similarly, there is insufficient information on how best to support people with sarcoma and assist individuals and carers to navigate changes in physical activity and manage nutrition requirements.

### 1.1 Aim

To explore the physical activity and nutritional supportive care needs of people living with sarcoma from the perspectives of people with sarcoma, their carers, and HCPs.

## 2 Method

### 2.1 Design

This study was part of a wider program of research examining information and support needs of people with sarcoma and their carers to inform the development of a sarcoma website [21] and a telephone helpline [22] to address their unmet information needs. Initial data analysis for the larger research program identified the need for information and support around physical activity and nutrition. This initiated a detailed analysis to understand the physical activity and nutritional supportive care needs of people living with sarcoma from the perspectives of people with sarcoma, their carers, and HCPs.

An exploratory qualitative research design grounded in interpretivist epistemology was used. A phenomenological approach was employed to provide a lens for understanding the lived experiences of people with sarcoma focusing on describing what physical activity and nutritional needs they experienced following diagnosis and through to recovery [23, 24, 25]. Semi‐structured interviews and focus groups were used to gain an understanding of participants' experiences from the perspectives of people with sarcoma and carers. We initially planned to conduct focus groups with each participant group; however, we found that it was difficult to schedule focus groups with times that worked nationally. We therefore opted to conduct interviews with participants who were unable to attend scheduled focus groups.

The perspectives of HCPs captured during semi‐structured interviews were included in our analysis to understand their experiences of discussing exercise and nutrition with people with sarcoma and their caregivers and inform the current clinical landscape and provide recommendations for supporting the exercise and nutrition needs of people with sarcoma and their carers. Multiple perspectives were included to avoid role‐bounded findings or exclusively perspectives of people with sarcoma, as carers and HCPs play an integral role in supporting individuals throughout the experience [25, 26]. Other qualitative studies have similarly explored the perspectives of patients, carers and health professionals using phenomenology to understand different multi‐faceted phenomenon [27, 28].

### 2.2 Ethics

Ethics approval was gained from Curtin University Human Research Ethics Committee (HRE2023‐0009). This research was conducted in accordance with the Declaration of Helsinki.

### 2.3 Participants

Three groups were interviewed: people with sarcoma; carers (including bereaved carers); and, healthcare professionals (HCPs) involved in management of people with sarcoma. Eligibility criteria included: people > 16 years‐old, diagnosed with sarcoma in the past 15 years, and able to converse in English; carers of people diagnosed with sarcoma including parents, partners, and siblings > 16 years‐old; or, HCPs who had provided care for people with sarcoma in the previous 5 years.

### 2.4 Recruitment

Participants were recruited using convenience sampling via Facebook, LinkedIn, and Twitter/X between 02/02/2022‐20/04/2024. Sock it to Sarcoma! and Cooper Rice‐Brading Foundation assisted with recruitment by advertising to their members via flyers, newsletters, and social media. Passive snowball sampling was used to recruit additional participants. Individuals who contributed to previous projects and consented to be contacted about future research were invited to participate. Additionally, HCPs were recruited nationally using the researchers' networks, steering committee members, and through the Australian and New Zealand Sarcoma Association (ANZSA).

### 2.5 Procedure

Participants were interviewed using semi‐structured interviews or focus groups. Questions focused on individual's experiences of having sarcoma, their information needs, and desired resources to assist individuals and their carers navigate sarcoma. Carers were asked similar questions and additionally about their needs as a carer. An interview guide for each group (developed with consumer representatives) was used with prompts for exploration of individual meanings. Example interview questions included:

1. In your experience, what were some of your key information needs?
  Prompts included: at diagnosis, treatment, during survivorship, and palliative care
2. What information were you looking/reaching out for?
3. What information did you feel was missing?

If participants mentioned exercise or nutrition additional prompts like *‘Tell me more about…’* were used to explore their information and support needs relating to exercise and nutrition.

Due to scheduling conflicts, interviews replaced focus groups for all HCPs. During interviews, HCPs were asked their perspectives on the information and support needs of people with sarcoma. Interviews commenced with the following question: In your experience, what are some of the key support needs for patients diagnosed with sarcoma? Prompts: psychosocial needs, information needs, family support needs etc. Full interview guides for the larger study are available from the research team on request.

Interviews were completed by senior researcher G.K.B.H. (PhD, BMedRad(Hons), woman) and research assistant CF (BPsych(Hons), man) either face‐to‐face or via Microsoft Teams, depending on participant's preferences. Online focus groups were facilitated by two senior researchers G.K.B.H., M.O. (PhD, MSc, B Arts(Hons), woman).

Prior to commencing interviews and focus groups, all participants were provided the information sheet and provided informed consent before completing demographics questions using an online Qualtrics or paper‐based questionnaire. Participants consented to interview recordings.

### 2.6 Analysis

Interviews and focus groups were audio recorded, transcribed verbatim, and analysed using reflexive thematic analysis. Data were managed in NVivo version 12. Braun and Clarke's six phases for reflexive thematic analysis were used to derive themes [29]. Steps included familiarisation with the data, generating initial codes, searching for themes, reviewing themes, defining and naming themes, and producing the final manuscript. Initial thematic analysis focused on describing individual lived experiences of people with sarcoma and their carers. Interviews with healthcare professionals were then analysed to determine their perspectives of providing support around physical activity and nutrition to people with sarcoma.

Recruitment continued until information power was achieved for each participant group and no new meaning was being derived [30]. Information power focuses on gaining a detailed and rich understanding of what information and support individuals need about sarcoma.

### 2.7 Quality

Braun and Clarke's Thematic Analysis Checklist and the Consolidated Criteria for Reporting Qualitative Research (COREQ) guidelines were used to ensure the quality and rigour of the study [31, 32]. Field notes and an audit trail was kept throughout the project to record study processes and protocol changes. Additionally, several researchers (G.K.B.H., J.D., C.F.) analysed the data and identified themes to ensure a broad spectrum of interpretations was captured. The researchers focused on ensuring that the perspectives of people with sarcoma, carers and health professionals were captured throughout the manuscript and then used to inform the recommendations for practice [33]. Reflexive journaling was used by researchers to document their reactions to findings and allow exploration of researchers' reactions and bias.

## 3 Findings

Physical activity and nutrition were discussed by 18 people with sarcoma (14 female), median age 51.0 years (range 21–61); 10 carers (10 female), median age = 55.5 (range 45–59); 3 bereaved carers (3 females) median age 54.0 (range 41–62), and 15 HCPs (12 female). All participants resided in Australia (see Tables 1 and 2 for demographics). Quotes are denoted with the following abbreviations: Person with Sarcoma = PwS, Carer = C, Bereaved Carer = BC, and Health Professional = HCP.

**TABLE 1:** Patient, carers and bereaved carers demographic data.

	People with sarcoma (*n* = 18)	Carers (*n* = 10)	Bereaved carers (*n* = 3)
Age at interview (years)			
Mdn (IQR)	51.0 (41–56)	55.5 (51–59)	54.0 (48–58)
Min.‐max.	21–61	45–59	41–62
	***N* (%)**	***N* (%)**	***N* (%)**
Age at diagnosis (years)			
Adolescent (10–19)	2 (11)	9 (90)	2 (67)
Adult (25–40)	4 (22)	0 (0)	1 (33)
Adult (> 40)	12 (67)	1 (10)	0 (0)
Sex			
Female	14 (78)	7 (70)	3 (100)
Male	4 (22)	3 (30)	0 (0)
State			
WA	10 (56)	5 (50)	1 (33)
QLD	3 (17)	1 (10)	0 (0)
NSW	2 (11)	2 (20)	0 (0)
VIC	2 (11)	2 (20)	1 (33)
SA	1 (6)	0 (0)	0 (0)
ACT	0 (0)	0 (0)	1 (33)
Location			
Major city	13 (72)	9 (90)	3 (100)
Regional/rural	5 (28)	1 (10)	0 (0)
Sarcoma type			
Bone	6 (33)	8 (80)	2 (67)
Soft tissue	12 (67)	2 (20)	1 (33)
Carer role			
Parent	—	9 (90)	2 (67)
Spouse	—	1 (10)	1 (33)

**TABLE 2:** Healthcare professionals demographic data.

Healthcare professionals (*n* = 15)	*N* (%)
Sex	
Female	12 (80)
Male	3 (20)
State	
WA	10 (67)
SA	2 (13)
ACT	1 (7)
NSW	1 (7)
QLD	1 (7)
Location	
Major city	15 (100)
Profession	
Nurse	3 (20)
Medical oncologist	3 (20)
Care coordinator	3 (20)
Exercise physiologist	2 (13)
Counsellor	1 (7)
Occupational therapist	1 (7)
Physiotherapist	1 (7)
Surgical oncologist	1 (7)

Our interpretation of meaning across participants' individual experiences, resulted in the identification of five themes: *Physical impact of sarcoma*; *Uncertainty about looking after the person with sarcoma*; *Needing rehabilitation* *support; Creating a new norma*l; and *Physical activity and healthy diet promote psychosocial well‐being* (See Table 3).

**TABLE 3:** Themes and subthemes.

Themes and sub‐themes
Physical impact of sarcoma
Grieving loss of ability to participate in physical activity
Uncertainty about looking after the person with sarcoma
Uncertainty about participating in physical activity → information about physical activity
Uncertainty about diet and nutrition → managing lack of appetite and addressing nutritional needs
Needing rehabilitation support
Creating a ‘new normal’
Physical activity and healthy diet promote psychosocial well‐being

### 3.1 Physical Impact of Sarcoma

Invasive surgeries took a significant physical toll on many people with sarcoma:

It was a massive operation; it went for about 12 hours, and it was a scar from my sternum to my pubic bone…it took me a year to physically recover.

[PwS22]

It's really, really hard. You feel like you're 90 years of age… You need to be strong in the shoulders because I was full‐time on crutches.

[PwS15]

HCPs described impacts on physical functioning:

The other big physical issue, is the fact that sarcoma will often affect people's mobility or other physical functioning, depending on where their sarcoma might be.

[HCP22]

#### 3.1.1 Grieving Loss of Ability to Participate in Physical Activity

Many people with sarcoma expressed sadness when reflecting on their previous abilities and loss of capacity to engage in physical activity:

I would jump out of bed, put my sneakers on and be running 12 km in literally 15 minutes. Now I have to get up later because I can’t get up earlier, because I spend four times a night getting up to go to the toilet, catheterising.

[PwS18]

This included the impact of amputation:

Can't ride a bike! Can't you know? The whole thing was just like, wow. And it was like grieving.

[PwS15]

Individuals missed being able to enjoy sports, with carers also describing this:

It was so important he just absolutely had to get back to playing his sport.

[C3]

The change in physical functioning impacted individuals' vision of their lives and their family roles:

I was hoping when I had a grandchild that… I would introduce them to ice skating. It'd be me because I was the ice skater. I could still skate up until my amputation.

[PwS15]

Thus, the sarcoma experience impacts on physical capacity affected interactions with their families beyond treatment and throughout survivorship. In addition to the impact on physical ability, many participants discussed uncertainties around both physical activity and nutrition and their desire for guidance.

### 3.2 Uncertainties About Looking After the Person With Sarcoma

This theme is presented across two subthemes concerning uncertainties about physical activity and nutrition.

#### 3.2.1 Uncertainty About Participating in Physical Activity

Participants experienced uncertainty about how to participate in physical activity safely following surgery and treatment. One individual recalled: ‘What will happen?…[I can't] do things like I did.’ [PwS13]. Another person with sarcoma explained the uncertainty they experienced and information they needed:

When I was talking to him about what I’m doing, all these exercises, he’s going why?…I want to be able to walk properly again, I want to be able to ride a bike…kick a ball, all these other things…I was basically given the surgery, go home, when you get better, when it heals up, good luck. So many people have an operation… the surgeon will do the operation, but there’s no pre‐surgery activities, there’s no post‐surgery activities.

[PwS1]

Many individuals and carers received inconsistent recommendations about physical activity and appropriate exercises before and after surgery. For example, one carer explained:

One physio we went to gave some exercises that, when we showed the oncologist, the oncologist said ‘stop that immediately, do not do that anymore’…. [because] it was pushing on the tumour.

[C1]

HCPs explained there are many uncertainties for individuals following surgery:

What is their function going to look like… What their range of movement is going to be looking like, and what's the end goal. And when is the recovery going to be over basically.

[HCP14]

Another HCP explained:

[Patients ask] am I going to be paralysed or disfigured or not be able to walk or do things properly?.

[HCP19]

This uncertainty and lack of consistent information made post‐surgery recovery difficult. The type of sarcoma and treatment also impacted rehabilitation needs, as one HCP noted:

The other big physical issue, is the fact that sarcoma will often affect people's mobility or other physical functioning depending on where in their body their sarcoma might be.

[HCP22]

HCPs also highlighted physical capability changes impact on an individual's ability to return to work:

So, if they're construction workers, when are they going to be able to climb the stairs, things like that.

[HCP14]

Instead of providing general information about mobility and physical activity, HCPs observed there were many issues unique to sarcoma, necessitating sarcoma‐specific advice tailored to individuals:

There's issues around mobility that perhaps other cancers don't have. So, asking for that kind of modified support, being aware of things like Allied Health and that…the sarcoma journey can be quite unique…isolating…there's a lot of need for really accurate information and acknowledgement of how unique sarcoma is.

[HCP10]

#### 3.2.2 Information About Physical Activity

All participant groups highlighted the need for a comprehensive program for physical activity and rehabilitation to be provided before treatment. For example:

Just having those little bits of information. One of the things I said very early on was about exercise and getting fit, getting ready for this operation; I think that’s really important because it makes it easier for everybody involved.

[PwS6]

HCPs recommended information needs to be sarcoma‐specific and tailored to individuals:

There are issues around mobility that perhaps other cancers don't have. So, asking for that kind of modified support, being aware of things like Allied Health and that…the sarcoma journey can be quite unique…isolating…there's a lot of need for really accurate information and acknowledgement of how unique sarcoma is.

[HCP10]

HCPs reported information needs around function and range of movement:

[Describing] what is their function going to look like…What their range of movement is going to be looking like, and what's the end goal? And when is the recovery going to be over?.

[HCP14]

#### 3.2.3 Uncertainty About Diet and Nutrition

During treatment and recovery, carers were unsure of which foods to provide. One carer explained:

Is there any particular diet…[family member] is throwing up all the time from chemo and can’t keep any food down, what sort of food can I give them?.

[C3]

Some individuals and carers noted HCPs did not provide information about diet, even downplaying the role of nutrition, which increased their sense of uncertainty.

When I asked my oncologist how can I change my diet to help as some support of treatment. They said, ‘Oh no, there's nothing you can…Your diet’s not going to make a difference’.

[PwS7]

Others desired information around vitamins and supplements to support their well‐being and recovery.

I went to a professional to give me extra vitamins and supplements and also more time in preparing myself mentally for the journey.

[PwS19]

#### 3.2.4 Managing Lack of Appetite and Addressing Nutritional Needs

Managing diet and nutrition was complicated by treatment effects, loss of appetite, and quality of food available. One carer explained the importance of having food available when the person with sarcoma was hungry:

She was sometimes wanting to eat something, we couldn't get it to her fast enough to actually get that window of being able to eat something. And then she couldn't eat again. So, it's trying to work out the food thing. The hospital food….

[C1]

The quality of food within hospitals exacerbated these issues, making it difficult for people with sarcoma to eat. Carers went to great lengths to aid consumption of food, including taking their own food to the hospital and buying take‐away food, when required:

If it was just up to the hospital food, he wouldn’t eat. He lost a lot of weight and if he said I feel like a hamburger or I feel like some—because he lost his taste buds, anything spicy was amazing. If he said, I feel like a burrito, I’d walk 3 km and find him a burrito. Or if, he wanted Thai food, I’d find it…in the beginning I was like no, no, you need to eat healthy. But if he was doing that, he wasn’t eating at all. It was a weigh up between giving him what he wants to eat, so he’s eating versus him not eating at all.

[C17]

Parents and carers often resorted to taking their own food to the hospital to try to ensure that their child continued to eat:

I took our own food, every time we go in a hospital. So frozen meat, rice cooker, some veggies. So, I can cook something that [Name] wants to eat because she had no appetite and hospital food, hmm, no.

[C10]

A burden was placed on the family, as they felt they needed to provide their children with food in the hospital and address nutritional needs themselves. The quality of hospital food and the difficulties enticing their children to eat also had a financial impact:

He didn’t want the food; he wanted to eat what he needed to eat and so we spent a fortune on food, like thousands of dollars over the 10 months on food, because he needed to eat. He couldn’t eat the hospital food so often I would eat his hospital food, and he would eat take‐away.

[C17]

Managing diet and nutrition was complicated by treatment‐related issues, loss of appetite, and the diversity in quality of food available. People with sarcoma and carers expressed a need for more information and support to continue adequate nutrition during treatment. HCPs also observed individuals needed detailed information about nutrition tailored for supporting the person with sarcoma:

My patient feels sick. They don't want to eat a whole chunk of steak… so information about how to manage the kind of practical functionality.

[HCP2]

When asked about what information people with sarcoma wanted, one HCP explained:

Is there any particular diet I should be on when I'm having sarcoma treatment? …they should be directed back to the treating team who have access to dietitians…even if the team doesn't have any nutritionists or dietitians, then they can know that someone was interested in that and direct things accordingly.

[HCP4]

HCPs also observed that they needed to provide information about aspects of treatment that impacted nutrition and diet:

The little things around that disease, the eating, the diet and the constipation… So, it is just a little thing that is a big thing, you know? It is a big thing for the person that is constipated.

[HCP10]

### 3.3 Needing Rehabilitation Support

After treatment, individuals and carers wanted information on access to support and services for physical rehabilitation:

I think that’s an aspect that needs to be looked into, making sure that rehab and recovery services are available, and who your point of call is. Even if you have a rehab case manager assigned to you, or something would be really good.

[BC8]

Individuals did not have access to HCPs or equipment to assist them with rehabilitation:

I wasn’t given a cane. I couldn’t walk. And I didn’t really get any rehabilitation. No physiotherapists came near me. Those physiotherapists dealt with me in the hospital and then they never said to me, going forward, when you go home, the first week I don’t want you to walk. I don’t want you to walk for three weeks. Then I want you to walk 20 minutes a day, for three days. Nothing.

[PwS18]

HCPs also highlighted the importance of rehabilitation and linking individuals with the multidisciplinary team:

With the surgery and if they have an amputation or anything like that…then they’ve got to be linked in with a physiotherapist, the OT.

[HCP1]

Participants highlighted rehabilitation needed to be tailored to both their physical and mental health needs:

Even if they've given me better exercises, I was in no fit state for many months to do it.

[PwS16]

The emotional impact of sarcoma treatment required rehabilitation that accommodated evolving needs. Learning to adjust to changes in exercise tolerance and capabilities was an area that many expressed needing more support pre‐ and post‐surgery. One HCP observed:

It's been well researched that prehabilitation does reduce length of stay, surgical site infection … and reduced pain post‐operatively. Exercising if you can, all those sorts of things….

[HCP19]

### 3.4 Creating a New ‘Normal’

Creating a new ‘normal’ and finding ways to adapt to changes was often difficult. A few described this slow process:

It took a lot of energy to build up strength and to do anything; a tiny little walk around my room would sap my energy for two hours. And then you would repeat the process.

[PwS14]

Chemotherapy and associated medical treatments also diminished muscle strength, making it difficult to mobilize:

I still struggle a lot with my mobility because, the muscles, I went straight into chemo and there was just no way of [building strength].

[PwS16]

Changes in routine and time requirements were necessitated by managing life after treatment. Individuals who had experienced amputation had specific requirements and needed support to regain mobility, as described by one HCP:

They may lose a limb, or they may have a limb prosthesis put in, so they may have difficulty in walking, they may have difficulty in arm movement. Mobility is one of the key things with this group of patients.

[HCP2]

Individuals experienced movement challenges, even with minor activity:

When it comes to [lower limb] amputations, the higher up the amputation, the more dependent you are on your shoulders and your arm strength, because I've lost the ability to sit up in bed to turn as well. I can't turn because you always use your leg as a counterbalance.

[PwS15]

Engagement in activity helped to foster a sense of normality. Individuals wanted to continue to exercise and find a way to do things:

And the life afterwards can still be walking, running, doing stuff I used to do before, albeit I just do it a little bit differently and a little bit slower. There are some activities which I won't do anymore, because I've got this metal in my leg and it only bends 90°, and I have had a couple of accidents. There's a lot of pain.

[PwS1]

Individuals needed to adapt previous exercises to accommodate their changed physical health, but recognized that, even if they were exercising differently, it was important to their psychosocial well‐being. Some HCPs described their efforts to support individuals with allied health referrals:

I try to get everybody to the exercise physiologist. Have some kind of exercise plan, and if they're completely new to exercise then I can refer to exercise physiology. If they have a little bit of knowledge of fitness and movement, then I really encourage that.

[HCP11]

### 3.5 Physical Activity and Healthy Diet Promote Psychosocial Well‐Being

This theme explored perceptions that physical activity and diet are beneficial to not only physical health, but psychosocial health and recovery. One individual described:

I think it [physical activity] really helps you up here [head], the body feels good, the brain feels it works together and that's something that I don't think it gets talked about the psychology part of healing.

[PwS15]

HCPs also linked mental and physical health:

Mental health and physical health [are connected], if they've had surgery, it's limitations around surgery, but even then, there's still stuff they can do. Still, go for a walk. And so they're the things I think would be really helpful for people.

[HCP11]

Individuals recognised links between diet and physical activity, adopting a broad, holistic view of health

The diets and lifestyle changes and things like that is really big. So, I'm going down the holistic metabolic approach, as well as the general.

[PwS5]

HCPs acknowledged relationships between health behaviours and subsequent impacts for well‐being:

I think from a bio‐psychosocial perspective their exercise regime, their diet, the whole thing about the microbiome and diet is so important for your mental health.

[HCP2]

## 4 Discussion

We explored the lived experiences of people with sarcoma and the perspectives of carers and healthcare professionals to understand what physical activity and nutrition unmet needs people living with sarcoma experience. Consistent with previous research [5], we found treatment effects meant individuals struggled to maintain healthy lifestyle and eating behaviours. People with sarcoma experienced challenges with adapting to changed physical activity capabilities and dietary needs. Our study has revealed gaps in high‐quality, consistent information about physical activity and nutrition for people with sarcoma. Consequently, people with sarcoma are at increased risk of adverse clinical outcomes including treatment toxicity, surgical complications, malnutrition, sarcopenia and poorer survival outcomes.

Sarcoma and treatment‐related changes to physical functioning were extreme and created a sense of loss and grief, altering or precluding physical and recreational activities for lengthy periods or indefinitely. Physical activity capabilities were central to individuals' identities and perceptions of their future selves. Similarly, people with mesothelioma report a loss of personal future and plans for family life, due to their inability to participate in activities [34].

The lack of consistent information on appropriate exercise and nutrition following sarcoma treatment exacerbated anxiety and uncertainty, and delayed individuals' recovery, including recovery of optimal mental health. Previous research highlights that people with cancer value advice from HCPs and require their support to foster motivation [35]. Additionally, social support can ameliorate support needs and encourage physical activity and healthy diet choices following a cancer diagnosis [36].

People with sarcoma wanted tailored physical exercise programs and nutritional advice to manage the physical impacts of treatment. Individualized programs with multidisciplinary team support, including physiotherapists and occupational therapists, were seen as necessary. We found those affected by sarcoma and their HCPs valued rehabilitation and exercise guidance prior to treatment, to improve physical and mental preparedness, enhance treatment outcomes, and establish the ‘new normal’. People with sarcoma should be offered tailored prehabilitation, an adapted exercise program in the hospital environment based on their psychobiological status, and a physical activity program during recovery [7, 37]. The benefits of physical activity and rehabilitation for people with osteosarcoma [38] and lower limb salvage surgery for sarcoma [39] are documented; however, further research is required to assess the effectiveness and practically implement exercise and rehabilitation programs for people with sarcoma. Exercise programs and rehabilitation may require flexibility depending on diagnosis and patient demographics [40].

Carers of adolescents (10–19 years) communicated the importance of continuing physical activities, where possible, and the need for nutritional advice throughout their child's sarcoma experience. Research has emphasized the value of physical activity during hospitalization post‐treatment of childhood cancer, and to have peers as motivators [41]. Motivation to exercise was linked to daily well‐being [41], suggesting physical activity support needs to be embedded into health systems, with follow‐up support for individuals and families to maintain motivation [42].

People with sarcoma and their carers communicated the importance of nutrition, but had not received information on eating healthily to assist treatment, recovery, and psychosocial well‐being. This aligns with research showing individuals with cancer are not provided with information to maintain nutrition [17, 18]. The nutritional needs of individuals receiving sarcoma treatment are complex, necessitating screening for malnutrition and assisting individuals to maintain a healthy diet during and after their sarcoma journey [16]. A recent scoping review on diet in adolescent and young adult patients highlighted the potential benefit of providing individualised nutrition counselling via telehealth and digital technologies, and recommended further measurement and implementation of dietary interventions for young people [43].

People with sarcoma articulated multiple benefits of physical activity and nutrition, including improving their psychological well‐being. These findings are consistent with a recent systematic review demonstrating tailored interventions, including physical activity, nutrition, psychosocial change, and symptom management, can improve QoL for cancer survivors [44]. Tailored lifestyle interventions should include behaviour change strategies, such as education, counselling, feedback, and monitoring [45, 46]. In conjunction, self‐determination can support intrinsic motivation to initiate and maintain exercise following a cancer diagnosis [47]. Further research should develop interventions to optimally motivated individuals to improve physical activity, nutrition, and psychosocial well‐being after sarcoma.

There remains a need for a comprehensive and coordinated support system to integrate exercise, rehabilitation and nutritional supportive care into treatment pathways, supporting uptake and adherence to health programs for people with sarcoma. This includes screening for unmet needs, such as physical activity and nutrition, structured and timely referral processes, clear clinical guidance, and ongoing multidisciplinary input. Physical activity and nutrition interventions must be consistently implemented and sustained across the cancer care continuum for sustained cancer‐related health outcomes.

### 4.1 Limitations

Interviews were conducted as part of a larger study on information and support needs and did not specifically ask about physical activity or nutrition, which may have yielded more in‐depth insights. Additionally, purposive sampling may have resulted in self‐selection bias and those who were not engaged with the sarcoma support organisations may not have participated. Many carers in this study were parents, rather than spouses, with a higher proportion of females in each of the participant groups. Given this study was embedded within a broader research program focused on the development of a sarcoma website [21] and telephone helpline [22], the HCP sample did not include a dietitian or nutritionist with expertise in sarcoma care. This may have limited our collection of data relating to diet and nutrition, and our ability to make specific nutrition‐related recommendations.

### 4.2 Clinical Implications

Provision of information and support around physical activity and nutrition should account for adjustments for individuals both during and post treatment. Table 4 provides a summary of themes and subthemes and recommendations based on our results and international recommendations for exercise [48], nutrition [16] and rehabilitation [50, 51] for people with cancer.

**TABLE 4:** Summary of themes and subthemes linked with recommendations for practice (based on our results and international recommendations).

Themes and subthemes	Recommendations for practice
Physical impact of sarcoma	Provide support to people with sarcoma as they learn about the physical impact of sarcoma on their lives. Health behaviour resources and models of care are needed to support people with sarcoma throughout treatment and recovery. Exercise must be embedded into standard cancer care and referral to an accredited exercise physiologist or physiotherapist with experience in cancer care is recommended [48].
Grieving the loss of ability to participate in physical activity	
Uncertainty about looking after the person with sarcoma	Provide information about physical activity. Inform patients and carers about what to expect related to appetite and eating behaviours during treatment. Early involvement of multidisciplinary teams is required to promote physical activity and nutrition [16, 48]. Individualized physical activity and nutrition plans should be co‐developed with individuals and carers following a sarcoma diagnosis [49]. Multidisciplinary plans should be developed pre‐operatively and during treatment, tailored to individual physical abilities, fitness levels, and nutritional supportive care needs, to optimize long‐term outcomes [16, 48].
Uncertainty about participating in physical activity	
Uncertainty about diet and nutrition	
Needing rehabilitation support	Provide people with sarcoma with ongoing support to increase motivation and self‐efficacy to engage in rehabilitation and physical activity during and beyond cancer treatment. Rehabilitation for people with cancer should include: Range of motion and muscle‐strengthening exercises, balance, mobility and fitness training, and self‐directed exercise [50, 51] Training in the use of mobility aids [48] Advice on diet, nutrition, and maintenance of health behaviours [48].
Creating a ‘new normal’	Physical activity and nutritional supportive care for individuals and carers must be ongoing [16, 48]. People with sarcoma may need support to adapt and accommodate changes to their physical health and abilities to create a ‘new normal’.
Physical activity and healthy diet promote psychosocial well‐being	Psychosocial support is an essential component of multidisciplinary care and should harness the expertise of allied healthcare professionals, including accredited exercise physiologists, physiotherapists, occupational therapists, psychologists, and dietitians.

## 5 Conclusion

This study identifies physical activity and nutrition information and support needs for people with sarcoma. Multidisciplinary teams must provide ongoing physical activity and nutrition care from diagnosis and throughout survivorship to improve adjustment, health outcomes, and QoL for people with sarcoma. Tailored, sarcoma‐specific health behaviour interventions focused on physical activity, nutrition, and psychosocial well‐being must be developed, assessed and incorporated into multidisciplinary cancer care.

## Funding

This project was funded through Cancer Australia's Supporting People with Cancer Grant initiative. The content is solely the responsibility of the grant recipient and does not necessarily represent the official views of Cancer Australia. Professor Georgia Halkett was a recipient of a Cancer Council WA Research Fellowship. Dr Joanna Fardell is a Maridulu Budyari Gumal (SPHERE) Cancer CAG Senior Research Fellow and is supported by a Cancer Institute NSW Research Capacity Building Grant (2021/CBG003) and Abbie Basson Sarcoma Foundation Ltd. (Sock it to Sarcoma!).

## Conflicts of Interest

Mandy Basson, Executive Director of The Abbie Basson Sarcoma Foundation Ltd. Sock it to Sarcoma! declares she has no conflicts of interest relevant to this publication. Tania Rice‐Brading, Co Founder of the Cooper Rice‐Brading Foundation, declares that she has no conflicts of interest relevant to this publication. Mandy Basson and Tania Rice‐Brading have not received financial support, research funding, consultancy fees, honoraria, or any other benefits from commercial or industry entities that could influence the work presented. No personal, academic, or professional relationships exist that could be perceived to affect the impartiality of this manuscript. The remaining authors declare that the research was conducted in the absence of any commercial or financial relationships that could be construed as a potential conflict of interest.

## Data Availability

The data that support the findings of this study are available on request from the corresponding author. The data are not publicly available due to privacy or ethical restrictions.
